# Weighted SNP Set Analysis in Genome-Wide Association Study

**DOI:** 10.1371/journal.pone.0075897

**Published:** 2013-09-30

**Authors:** Hui Dai, Yang Zhao, Cheng Qian, Min Cai, Ruyang Zhang, Minjie Chu, Juncheng Dai, Zhibin Hu, Hongbing Shen, Feng Chen

**Affiliations:** 1 Department of Epidemiology and Biostatistics, School of Public Health, Nanjing Medical University, Nanjing, China; 2 Section of Clinical Epidemiology, Jiangsu Key Laboratory of Cancer Biomarkers, Prevention and Treatment, Cancer Center, Nanjing Medical University, Nanjing, China; 3 State Key Laboratory of Reproductive Medicine, Nanjing Medical University, Nanjing, China; Children's National Medical Center, Washington, United States of America

## Abstract

Genome-wide association studies (GWAS) are popular for identifying genetic variants which are associated with disease risk. Many approaches have been proposed to test multiple single nucleotide polymorphisms (SNPs) in a region simultaneously which considering disadvantages of methods in single locus association analysis. Kernel machine based SNP set analysis is more powerful than single locus analysis, which borrows information from SNPs correlated with causal or tag SNPs. Four types of kernel machine functions and principal component based approach (PCA) were also compared. However, given the loss of power caused by low minor allele frequencies (MAF), we conducted an extension work on PCA and used a new method called weighted PCA (wPCA). Comparative analysis was performed for weighted principal component analysis (wPCA), logistic kernel machine based test (LKM) and principal component analysis (PCA) based on SNP set in the case of different minor allele frequencies (MAF) and linkage disequilibrium (LD) structures. We also applied the three methods to analyze two SNP sets extracted from a real GWAS dataset of non-small cell lung cancer in Han Chinese population. Simulation results show that when the MAF of the causal SNP is low, weighted principal component and weighted IBS are more powerful than PCA and other kernel machine functions at different LD structures and different numbers of causal SNPs. Application of the three methods to a real GWAS dataset indicates that wPCA and wIBS have better performance than the linear kernel, IBS kernel and PCA.

## Introduction

At present, genome-wide association study (GWAS) has been a popular approach for studying the genetic susceptibility of complex diseases. Nowadays, chips used in GWAS can simultaneously scan hundreds of thousands or even more SNPs in comparatively wide chromosomal regions by comparing the frequencies of genetic variants in cases and controls and estimating whether the locus is associated with the disease [1], [2]. Association tests can be generally classified into two aspects: single locus association tests and multiple loci association tests [3]. It is common to run single locus association tests in the whole GWAS for identifying causal single nucleotide polymorphisms (SNPs) with strong effects on disease. However, such a SNP-wise analysis may result in computational burden and the well-known issue of multiple testing [4]. A multiple testing adjustment procedure is usually required to ensure the overall type I error rates remain at an acceptable level, such as Bonferroni correction [5], [6] and false discovery rates (FDR) [7]–[9]. As an example, when examining the effects of 500,000 SNPs in a GWAS, each test has to be conducted at the α = 10^−7^ level, and it is very stringent [10].

It is reported that complex diseases are caused by causal SNPs with weak effect (OR≤1.5) [11]. Recent studies suggest that the test power of existing methods is low after correction for multiple testing. For example, assuming that OR = 1.5, a GWAS including 600,000 loci has to recruit 1890 cases and 1890 controls to achieve a test power of 80% when MAF is 0.4. However, when MAF is close to 0.1, 4410 individuals are required in order to reach a test power of 80% [12], [13].

Test power can be improved if multiple SNPs are tested together which are associated in biology. Wu et al. applied logistic kernel machine to case-control GWAS to test for the SNP-set effect [10]. The result of Wu was that the kernel machine based SNP set analysis has greater power than single SNP analysis. But they didn't compare kinds of logistic kernel machine functions specifically. Gauderman et al. proposed a principal component based approach (PCA) which computed principal components (PCs) from SNP set and PCs were included in the regression model to test for the association [14]. Zhao et al. [15] compared four types of kernel machine functions with principal component based approach (PCA). Their study demonstrated that these methods are not powerful when the MAF is low (<0.2). The present work is an extension of Zhao et al. in which we aim to identify whether weighted SNP set analysis (including PCA and LKM) may increase the statistical power in the case of low minor allele frequencies (MAF) and different linkage disequilibrium (LD) structures.

In this article, the structure of comparing performances of wPCA, LKM and PCA by using simulated datasets is as follows. Firstly, the procedures of LKM, PCA will be briefly described and we introduce weighted PCA in detail. Secondly, results of several simulated simulation studies are provided to compare type I error rates and test powers of these methods. We then apply these methods to two SNP sets extracted from a real Lung Cancer GWAS data. At last, the article will end with a discussion section.

## Methods

### Ethics statement

This collaborative study was approved by the institutional review boards of China Medical University, Tongji Medical College, Fudan University, Nanjing Medical University and Guangzhou Medical College with written informed consent from all participants.

### Logistic Kernel Machine Based Test (LKM)

We assume that we have a SNP set including *p* SNPs from *n* individuals. Let 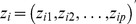 denote the genotypes of the *i*th individual. The disease outcome is denoted by *D* (1 = affected, 0 = unaffected).

For the *i*th individual, we have the semiparametric model given by
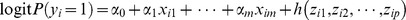
Where 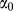 is an intercept term, 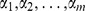 are regression coefficients corresponding to the environmental and demographic covariates. The SNPs, 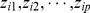, influence the disease outcome through the general function 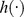, which is defined by 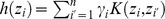 for some 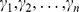. 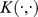 is a kernel function that measures the similarity of 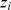 and 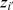 [15]. 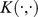 could be the linear, identical-by-state(IBS), weighted IBS [10]. The weighted IBS kernel is an extension of the IBS kernel that up-weights for similarity in rare alleles. In this article, we apply the weights based on *β* distribution proposed by Wu and Lee et al. [16]. The weight is taken as 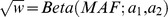 for a certain SNP. 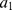 = 1, 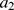 = 25, *Beta*() is the density function of *β* distribution.

Liu et al. provided the connection between LKM and generalized linear mixed model (GLMM) [17]. They showed that 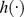 could be an arbitrary function with mean zero and variance 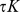, thus a score test with 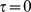 could be applied to test the null hypothesis of no association [15].

### Principal Component Based Analysis (PCA)

We use 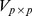 to denote the variance-covariance matrix of the SNP set, and 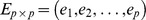 denotes the *p p*-dimension eigenvectors of 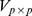. Let 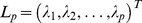 denote the *p* corresponding eigenvalues with 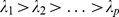 [15]. The principal components are defined by
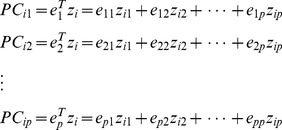

*e_i_* is selected to maximize the variance of PC*_i_*, and the constraint is 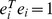. The covariance between PC_i_ and PC_j_ is 0 for arbitrary 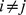. 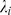 measures the variation which is explained by PC*_i_* and equals to its variance. Instead of using the *p* SNPs, we only need to select the first *k* PCs in which cumulative contribution 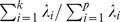 is greater than the threshold (eg. 80%). Therefore, we will just use the first *k* PCs in the multiple logistic model [15]

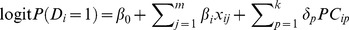



To test the significance of the SNP set, we can use a *k*-df likelihood ratio test. In our study, PCA (80%) is used to denote the PCA with the PCs explaining *Z*%(80%) of the total variation with the definition of 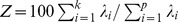.

### Weighted Principal Component Based Analysis (wPCA)

We propose a weighted principal component analysis. Let 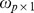 denote *p*-dimension weighted eigenvectors corresponding to *p* SNPs in the SNP set. So we use 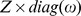 instead of Z in the extraction of principal components. 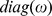 represents the diagonal matrix in which the diagonal elements are 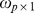 and others are 0.

The choice of weights is various, such as reciprocal of MAF or the important significance of SNPs in biology and so on. In this article, we apply the weights based on *β* distribution proposed by Wu and Lee et al. [16]. The weight is taken as 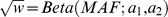 for a certain SNP. MAF is the minor allele frequency of this SNP, 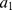 = 1, 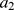 = 25, *Beta*() is the density function of *β* distribution.

### Data simulation

We use simulated datasets to compare the performances of wPCA, LKM and PCA. Measurements include empirical type I error rate and test power. We assume that all the causal SNPs can improve the risk. The disease model is assumed
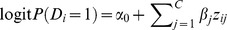
By definition, *C* denotes the number of causal SNPs. We set C = 0, 1 or 2 in our simulations which represents null model, single causal SNP model or two causal SNPs model. *j* represents the causal SNP, and 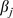 is the effect of the causal SNP.

#### The generation of simulated datasets

Simulated datasets are generated via cutting the random deviates sampled from multivariate normal distribution with specified correlation coefficient matrix [18]. And the simulated datasets are also checked to evaluate whether the generated MAF, LD structures of the simulated datasets are consistent with parameter values assigned before.

#### Simulations based on virtual datasets with single SNP set

To compare the wPCA, LKM and PCA, we apply a statistical simulation based on our simulated datasets (simulated datasets based on different MAF and LD structures) under the null hypothesis (H_0_) and alternative hypothesis (H_1_). We set two SNP sets, respectively. One is formed by 20 SNPs and the other includes 100 SNPs. Parameters of the virtual simulations are described by **Table 1**. Scenarios are set in three different MAFs (MAF = 0.2 for all SNPs; MAF = 0.04 for arbitrary one SNP and MAF = 0.1 for others; MAF = 0.04 for arbitrary two SNPs and MAF = 0.1 for others) and three LD structures (R^2^ = 0.1, 0.5 or 0.8 for any two SNPs). 1,000 cases and 1,000 controls are generated.

**Table 1:** Parameter settings of virtual datasets.

Scenario	MAF	LD	OR
A1	0.2	0.1/0.5/0.8	1.0
A2	0.04;0.1	0.1/0.5/0.8	1.0
A3	0.04;0.04;0.1	0.1/0.5/0.8	1.0
A4	0.1	0.1/0.5/0.8	1.2
A5	0.2	0.1/0.5/0.8	1.2
A6	0.04;0.1	0.1/0.5/0.8	1.2
A7	0.1	0.1/0.5/0.8	1.2;1.2
A8	0.2	0.1/0.5/0.8	1.2;1.2
A9	0.04 ;0.04;0.1	0.1/0.5/0.8	1.2;1.2

Scenarios A1–A3 are simulated to evaluate the performances of the three methods on controlling type I error under the null disease model(C = 0) where the outcome is independent of the loci. We calculate the empirical type I error rate as the proportion of rejecting the null hypothesis in the 2,000 simulated datasets. Scenarios A4–A6 are simulated to compare the powers of wPCA, LKM and PCA when there is only one causal SNP in the SNP set. We set the odds ratio (OR) as 1.2 at scenarios A4–A6. In all the three scenarios, any of the SNPs in the SNP set has the opportunity to be the causal SNP. We also set two causal SNPs in scenarios A7–A9 to compare the power of the three methods. The odds ratios of two causal SNPs are both 1.2. For scenarios A4–A9, 1,000 datasets are simulated. We calculate the test power as the proportion of *p*-values less than 0.05. All of SNPs in the SNP set are set as the genotyped SNPs.

#### Simulations based on the *CLPTM1L* gene

We simulate datasets on the basis of the *CLPTM1L* gene. *CLPTM1L*, encoding cleft lip and palate transmembrane protein 1-like protein, is a 27.35 kb-long-gene located at 5p13.33. In this gene, rs31489 and rs401681 were reported to be associated with non-small cell lung cancer (NSCLC) [19], [20]. The phased haplotypes of CHB (Han Chinese in Beijing, China) samples are downloaded from the HapMap web site (Phase 2, release 24). There are 28 SNPs locates within the range including ±20 kb of the *CLPTM1L* gene.

We conduct 8 scenarios of simulations based on the *CLPTM1L* gene (scenarios B1–B8). In scenario B1, 2,000 datasets are simulated with no association between the disease outcome and SNPs. In scenario B2, each of the 28 SNPs in the SNP set is set to be the causal SNP in turn with OR = 1.2, and 1000 datasets are simulated. To make the simulations more realistic, only 8 of the 28 SNPs, which are directly genotyped by the Illumina 610k Quad chip, are used by the three methods.

We also examine the ability of these methods from multiple causal loci in the case of 2 causal SNPs with OR = 1.2 in the SNP set (scenarios B3 to B8). In scenario B3, both of the two causal SNPs are genotyped. Only one of the two causal SNPs is genotyped in scenarios B4, B7 and B8. In scenario B5–B6, no causal SNPs are genotyped. Besides, the MAFs of the two causal SNPs are low in scenario B6. Just one MAF of the two causal SNPs is low in B7–B8. Details of these scenarios are presented in **Table 2**.

**Table 2:** Parameter settings of the *CLPTM1L* gene.

Scenario	Number of	Locations of the causal	Minor allele	Odds ratio
	causal SNPs	SNPs	frequency(MAF)	
B1	0	-	-	1.0
B2	1	1 of 28 SNPs in turn	-	1.2
B3	2	17 and 25	0.2 and 0.4	1.2
B4	2	17 and 6	0.2 and 0.067	1.2
B5	2	11 and 6	0.267 and 0.067	1.2
B6	2	8 and 9	0.144 and 0.189	1.2
B7	2	17and 8	0.2 and 0.144	1.2
B8	2	25 and 9	0.4 and 0.189	1.2

### Application of wPCA, LKM and PCA to a real GWAS dataset

We apply the three methods to a real GWAS dataset studying the genetic susceptibility of non-small cell lung cancer (NSCLC). The details of the population were described previously [20]. This dataset includes 1,473 NSCLC cases and 1,962 controls. DNA was extracted from the whole blood and genotyped by the Affymetrix 6.0 Quad chip. A total of 570,373 SNPs pass the general quality control (QC) [20]. We extracted two regions from the dataset. One is a region of 67 kb in 5p13.33, which includes 8 SNPs within a range of 20 kb upstream and downstream of the *CLPTM1L* gene, and the MAFs of 4 SNPs are lower than 20%. The gene was reported to be associated with smoking behavior and NSCLC [19]–[21]. The second region is about 208.4 kb length in 6p21.32–21.33 including 15 SNPs with genes of TNXB, FKBPL and PPT2, and the MAFs of 12 SNPs are lower than 20%. PPT2 was associated with pulmonary function [22] and gene expression of TNXB was reported to be associated with lung squamous cell cancer [23]. FKBPL has been proposed as a novel prognostic and predictive biomarker [24]. The two regions are then analyzed by wPCA, LKM and PCA, respectively.

Datasets are generated using R packages (version 2.13.0) and PLINK. Analyses of the simulated datasets are performed using R packages. The SKAT package is used to conduct LKM analysis.

## Results

### Simulations based on virtual datasets with single SNP set

#### Empirical type I error rate

**Figure 1:**
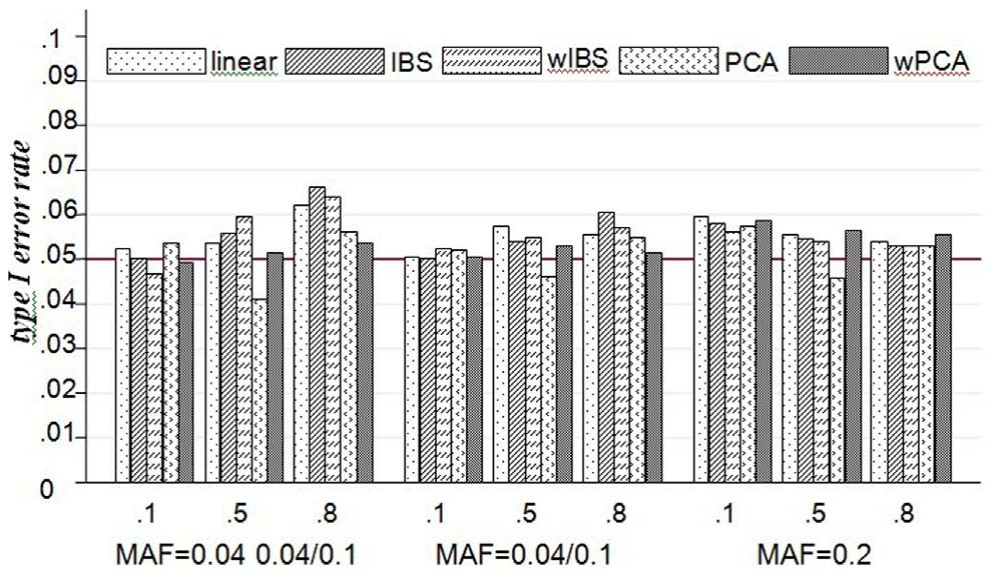
Empirical type I error rates for LKM, PCA and wPCA in scenarios A1–A3. The plot shows the empirical type I error rates (y-axis) based on virtual datasets of each method over the different LD and MAF structures (x-axis) with 20 SNPs. The first line of x-axis represents LD, and the bottom line is MAF.

The empirical type I error rates of LKM, PCA and wPCA are presented by **Figure 1**. All of the three methods control the type I error at the significance level of 0.05. For wPCA and PCA, the type I error rates are independent of the number of PCs and different weights included in the model.

#### Empirical test power based on virtual datasets with single causal SNP

**Figure 2:**
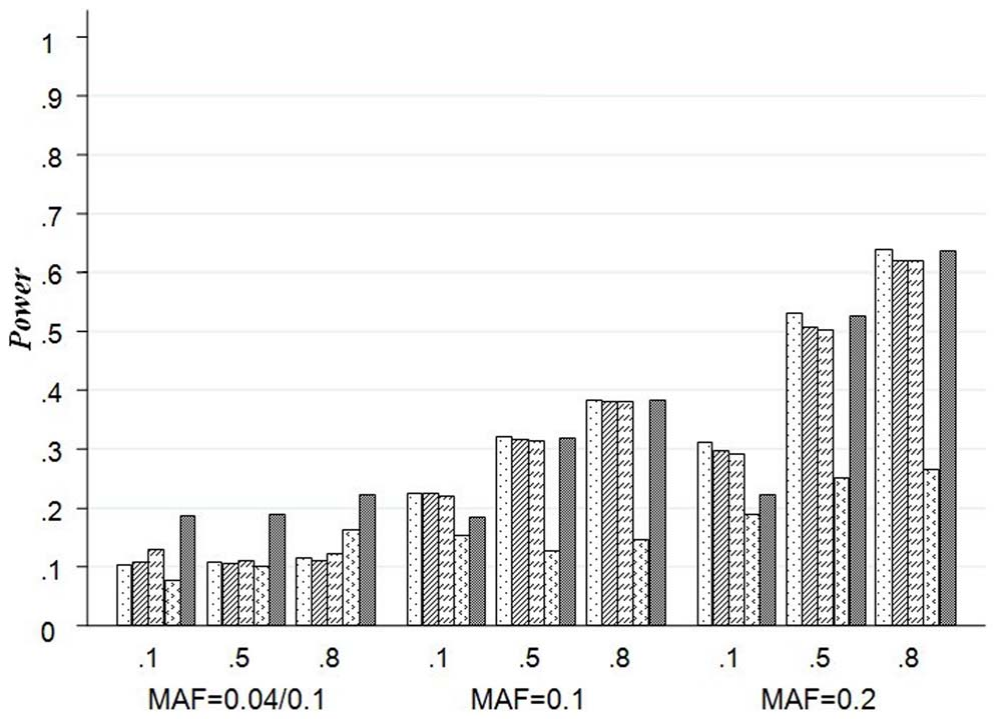
Test of Power for LKM, PCA and wPCA in Scenarios A4–A6. The plot shows the powers (y-axis) based on virtual datasets with single causal SNP of each method over the different LD and MAF structures (x-axis) with 20 SNPs. The first line of x-axis represents LD, and the bottom line is MAF.

Results from the simulation on scenarios A4–A6 are presented by **Figure 2**. On the basis of **Figure 2**, we can examine how the test power of each method varies with minor allele frequency (MAF) and LD structures. When the causal SNP has high MAF, the result of wPCA is similar with PCA. It is worth noticing that wPCA and LKM with wIBS are always much more powerful than the other methods when the MAF of the causal SNP is low. For example, with R^2^ = 0.8 of arbitrary two SNPs and MAF = 0.04 of causal SNP in A6, the power of LKM with linear kernel is 11.4% while the power of wIBS is 12.3% and wPCA is 22.2%. And also as an example, the power of IBS kernel is 10.6% (the greatest power of PCA and LKM except wIBS kernel) while the power of wIBS is 13.0% and wPCA is 18.6% with R^2^ = 0.1 of any two SNPs and MAF = 0.04 of causal SNP in A6.

#### Empirical test power based on virtual datasets with two causal SNPs

**Figure 3:**
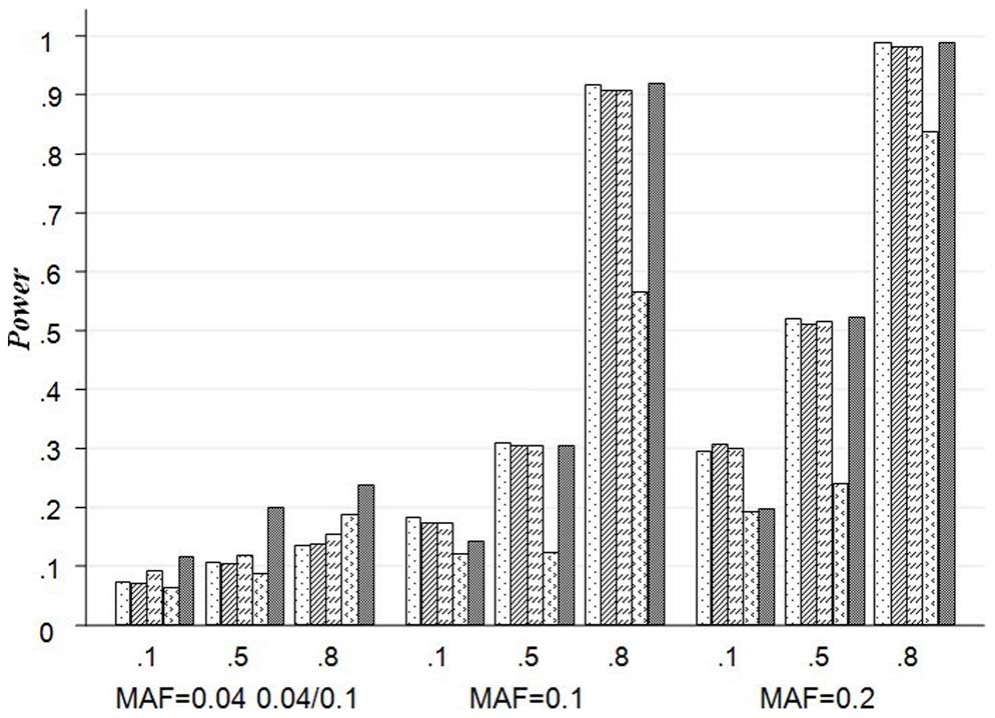
Test of Power for LKM, PCA and wPCA in Scenarios A7–A9. The plot shows the powers (y-axis) based on virtual datasets with two causal SNPs of each method over the different LD and MAF structures (x-axis) with 20 SNPs. The first line of x-axis represents LD, and the bottom line is MAF.

We present the results from scenarios A7 to A9 by **Figure 3**. Once again, the power is affected by the LD between the causal and genotyped SNPs and minor allele frequency (MAF). It is also interesting to find that LKM with wIBS and wPCA are more superior than the other methods in scenario A9 as the MAFs of both causal SNPs are low. For example, when the R^2^ = 0.1 for any two SNPs, the powers of the wIBS (9.1%) and the wPCA (11.6%) are much greater than other methods.

If both the causal SNPs are in strong LD with the other SNPs (0.8 for the R^2^ of arbitrary two SNPs) and relatively high MAF (MAF = 0.1 or 0.2), then most of these methods have test power greater than 90%. PCA and LKM with linear kernel are more powerful than the others. The results of type I error rate and test power of 100 SNPs in a SNP set are similar with that of 20 SNPs in a SNP set, the detail results are listed in **Figure S1 (in File S1)**, **Figure S2 (in File S1)** and **Figure S3 (in File S1)**.

### Simulations based on the *CLPTM1L* gene

#### Empirical type I error rate

The empirical type I error rates of LKM, PCA and wPCA are presented by **Table 3**. All of the three methods control the type I error at the significant level of 0.05 just as the empirical type I error rates based on virtual datasets.

**Table 3:** Empirical type I error rates for LKM, PCA and wPCA in Scenarios B1.

		LKM		PCA	wPCA
	Linear	IBS	wIBS		
α	0.0480	0.0550	0.0545	0.0485	0.0555

#### Empirical test power with single causal SNP

**Figure 4:**
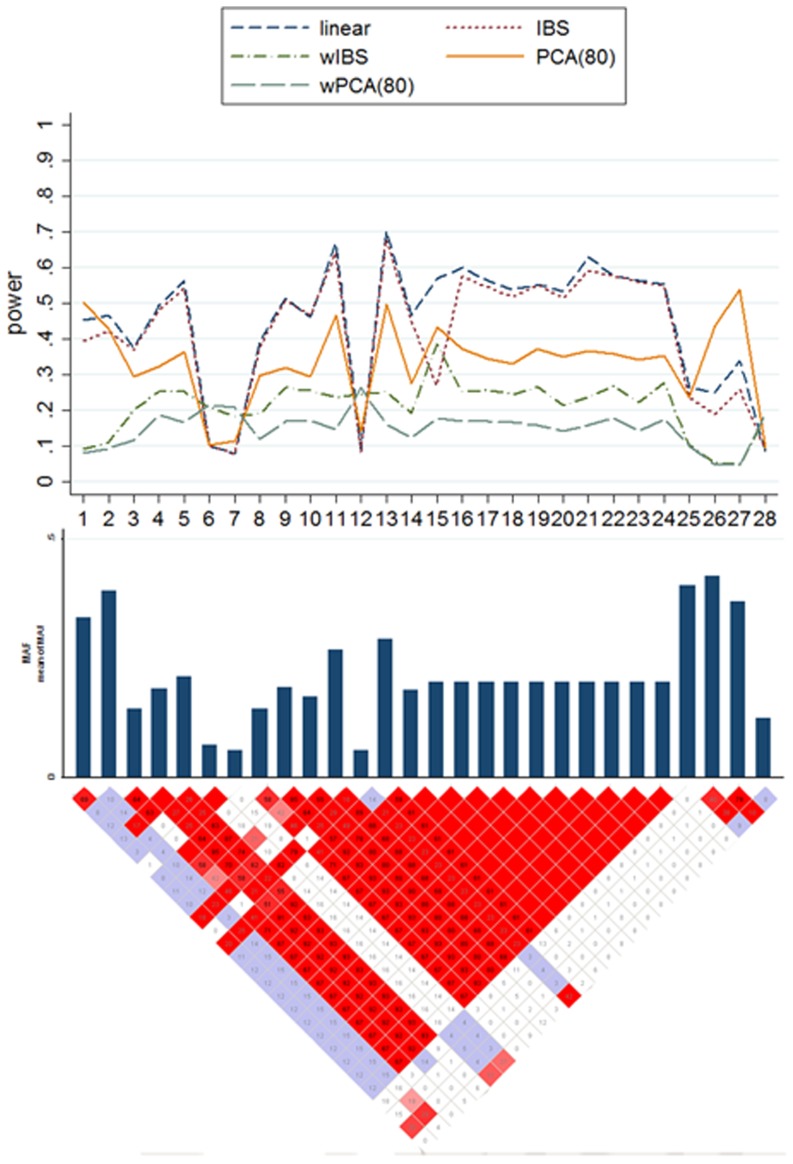
Test of Power for LKM, PCA and wPCA in Scenarios B2. The top plot shows the power (y-axis) of each method over the locations (x-axis) of the causal SNPs. The bar-plot shows the MAFs of all SNPs. The bottom plot shows the LD structure of the 28 SNPs downloaded from the HapMap project, in which the red scale indicates the value of r^2^ (1 = red, 0 = white).

Results from the simulation on scenario B2 are presented by **Figure 4**. It is important that when the MAF of the causal SNP is low (the 6th, 7th, 12th or 28th SNP in **Figure 4**), wPCA and wIBS have greater power than the others in general. For the 12th SNP, the power of wIBS is 24.6% and wPCA is 26.3% with the power of other methods ranging from 7.5% to 14.3%. And for the 6th SNP, the powers of wIBS(20.8%) and wPCA(21.7%) are also more powerful than other methods.

#### Empirical test power with two causal SNP

We also present the results from scenarios B3 to B8 by **Table 4**. As above, the power is affected by the MAF and LD structures. In scenario B6, power of most of the methods is less than 50%, except the LKM with wIBS and wPCA, as the MAFs of both causal SNPs are lower than 0.1. And the test power of the LKM with wIBS and wPCA from scenarios B7 and B8 is as or just slightly lower than that of other methods when only one of the two causal SNPs has low MAF.

**Table 4:** Test of Power for LKM, PCA and wPCA in Scenarios B3–B8.

Scenario		LKM		PCA	wPCA
	Linear	IBS	wIBS		
B3	0.996	0.989	0.747	0.955	0.430
B4	0.991	0.992	0.792	0.958	0.495
B5	0.948	0.945	0.751	0.888	0.408
B6	0.229	0.222	0.615	0.376	0.635
B7	0.934	0.910	0.658	0.689	0.551
B8	0.750	0.745	0.656	0.584	0.555

If both of the causal SNPs are in strong LD with the genotyped SNPs and high MAFs (scenarios B3–B5) where the advantage of wIBS and wPCA couldn't be reflected and results in weak powers, so most of these methods, except LKM with wIBS and wPCA, have test power greater than 90%.

### Application of LKM, PCA and wPCA to a real GWAS dataset

The results of the analysis can be found in **Table 5**. For the first SNP set, the least *p*-value in the SNP set is 2.19E-4(1.75E-3 after the Bonferroni correction for the effective number of tests). The least *p*-value of the LKM is wIBS kernel (1.30E-3). The *p*-value of PCA is 1.25E-2. The *p*-value of wPCA is 7.01E-4. And for the second SNP set, the least *p*-value in the SNP set is 5.01E-4(7.51E-3 after the Bonferroni correction for the effective number of tests). The least *p*-value of the LKM is wIBS kernel (1.65E-3). The *p*-value of PCA is 7.18E-2 and the *p*-value of wPCA is 7.50E-3.

**Table 5:** Results of LKM, PCA and wPCA on the Analysis of a SNP set from a real GWAS dataset.

	Individual SNP test			LKM			*P*-value of
SNP set	The least	*p*-values for the SNP set*	Linear	IBS	wIBS	PCA	wPCA
	*p*-value in the SNP set						
1	2.19E-4	1.75E-3	4.36E-3	4.30E-3	1.30E-3	1.25E-2	7.01E-4
2	5.01E-4	7.51E-3	1.57E-2	5.97E-3	1.65E-3	7.18E-2	7.50E-3

* After Bonferroni correction for the effective number of test.

## Discussion

In our study, we compare the statistical properties of weighted principal component analysis, weighted and un-weighted logistic kernel machine based test, principal component analysis from three aspects: dummy data structure, real data structure generated based on the haplotypes downloaded from the International HapMap Project and application of LKM and PCA, wPCA to a real GWAS data on NSCLC. The results suggest that four methods can control the type I error and have the ability to test the association between the outcome and the SNP set. When the MAF of the causal SNP is low, weighted principal component and weighted IBS are more powerful than PCA and other kernel machine functions at different LD structures and different numbers of causal SNPs.

Studies have shown that analysis based on SNP set can make full use of messages of multiple loci which have high LD with causal SNPs when there is LD between causal SNPs and genotyped SNPs, leading to an improved test power. All of the three methods can divide genome-wide SNPs into SNP set which is biologically meaningful in different ways. On the basis of prior biological knowledge, SNP sets can be made which will lead to additional gains in power [10].

At present, linear kernel, IBS kernel and PCA are popular methods in genome-wide association studies. But the applications of the three methods are limited when the MAF of the causal SNP is low. Based on wPCA and wIBS, our studies suggest that SNP set based on weights can increase the test power when MAF is low. The SNP with low MAF is given high weight by setting appropriate weights and therefore the test power is improved. Before selecting the Lee weights, we have attempted some other weighting schemes, such as the reciprocal of MAF and the important significance of SNPs in biology. However, the results which are not shown in this article suggest that applying the Lee weights performs better than the other weighting schemes. This is the reason why we choose the Lee weights in the paper. The simulation studies demonstrate that the test power of wPCA is higher than linear kernel, IBS kernel and PCA when the MAF of the causal SNP is low, while wIBS is similar with wPCA [25].

We also simulate the situations of PCA and wPCA with extracting different principal components, and the results are similar with Zhao [15]. With extracting the principal component, we first extract the large variation loci. When the causal SNP has low MAF and weak LD with surrounding SNPs, information can only be inflected by latter principal components. Failure to include the PCs representing the causal SNPs or include too many principal components in the model will both decrease the statistical power. By using weighted PCA, the variance of the SNP with low MAF will be enlarged when the SNP is given high weight, which increases the probability of the SNP to be presented by the top principal components. On the other hand, less principal components are needed to explain sufficient proportions of total variation, which decreases the consumption of degree of freedom and increase the power.

There are several limitations in our study. First, more complicated situations, such as gene-gene interaction, are not included in our study. Second, more scenarios are needed to compare wPCA, LKM and PCA. Last, due to the limited availability of prior knowledge concerning genetic mechanism, how to combine the methods mentioned in our paper still remains a challenge for a special analysis. Further work to solve such problems will certainly be warranted.

## Supporting Information

File S1 **Figure S1: Empirical type I error rates for LKM, PCA and wPCA with 100 SNPs.** The plot shows the empirical type I error rates (y-axis) based on virtual datasets of each method over the different LD and MAF structures (x-axis) with 100 SNPs. The first line of x-axis represents LD, and the bottom line is MAF. **Figure S2: Test of Power for LKM, PCA and wPCA in Scenarios A4–A6 with 100 SNPs.** The plot shows the powers (y-axis) based on virtual datasets with single causal SNP of each method over the different LD and MAF structures (x-axis) with 100 SNPs. The first line of x-axis represents LD, and the bottom line is MAF. **Figure S3: Test of Power for LKM, PCA and wPCA in Scenarios A7–A9 with 100 SNPs.** The plot shows the powers (y-axis) based on virtual datasets with two causal SNPs of each method over the different LD and MAF structures (x-axis) with 100 SNPs. The first line of x-axis represents LD, and the bottom line is MAF.
(DOCX)
