# Supplementary material for: Weighted SNP Set Analysis in Genome-Wide Association Study
Source: PLoS One. 2013 Sep 30;8(9):e75897. doi: 10.1371/journal.pone.0075897 (PMC3786949; doi:10.1371/journal.pone.0075897)
Supplement: File S1 — Figure S1: Empirical type I error rates for LKM, PCA and wPCA with 100 SNPs. The plot shows the empirical type I error rates (y-axis) based on virtual datasets of each method over the different LD and MAF structures (x-axis) with 100 SNPs. The first line of x-axis represents LD, and the bottom line is MAF. Figure S2: Test of Power for LKM, PCA and wPCA in Scenarios A4–A6 with 100 SNPs. The plot shows the powers (y-axis) based on virtual datasets with single causal SNP of each method over the different LD and MAF structures (x-axis) with 100 SNPs. The first line of x-axis represents LD, and the bottom line is MAF. Figure S3: Test of Power for LKM, PCA and wPCA in Scenarios A7–A9 with 100 SNPs. The plot shows the powers (y-axis) based on virtual datasets with two causal SNPs of each method over the different LD and MAF structures (x-axis) with 100 SNPs. The first line of x-axis represents LD, and the bottom line is MAF. (DOCX) [file pone.0075897.s001.docx]

0

.01

.02

.03

.04

.05

.06

.07

.08

.09

.1

MAF=0.04 0.04/0.1

MAF=0.04/0.1

MAF=0.1

MAF=0.2

.1

.5

.8

.1

.5

.8

.1

.5

.8

.1

.5

.8

t***ype I error rate***

linear

IBS

wIBS

PCA

wPCA

**Figure S1: Empirical type I error rates for LKM, PCA and wPCA with 100 SNPs**

The plot shows the empirical type I error rates (y-axis) based on virtual datasets of each method over the different LD and MAF structures (x-axis) with 100 SNPs. The first line of x-axis represents LD, and the bottom line is MAF.

0

.1

.2

.3

.4

.5

.6

.7

.8

.9

1

MAF=0.04/0.1

MAF=0.1

MAF=0.2

.1

.5

.8

.1

.5

.8

.1

.5

.8

***Power***

**Figure S2: Test of Power for LKM, PCA and wPCA in Scenarios A4-A6 with 100 SNPs**

The plot shows the powers (y-axis) based on virtual datasets with single causal SNP of each method over the different LD and MAF structures (x-axis) with 100 SNPs. The first line of x-axis represents LD, and the bottom line is MAF.

0

.1

.2

.3

.4

.5

.6

.7

.8

.9

1

MAF=0.04 0.04/0.1

MAF=0.1

MAF=0.2

.1

.5

.8

.1

.5

.8

.1

.5

.8

***Power***

**Figure S3: Test of Power for LKM, PCA and wPCA in Scenarios A7-A9 with 100 SNPs**

The plot shows the powers (y-axis) based on virtual datasets with two causal SNPs of each method over the different LD and MAF structures (x-axis) with 100 SNPs. The first line of x-axis represents LD, and the bottom line is MAF.
